# Neuroprotective mechanisms of Thai traditional brain tonic Phy-Blica-O against LPS-induced neuroinflammation: Inhibition of NF-κB in microglia and mice

**DOI:** 10.1371/journal.pone.0352429

**Published:** 2026-06-26

**Authors:** Thammarat Kaewmanee, Palanivel Ganesan, Piyapong Choochana, Pinanong Na-Phatthalung, Samuel Abiodun Kehinde, Sasitorn Chusri

**Affiliations:** 1 Department of Food Science and Nutrition, Faculty of Science and Technology, Prince of Songkla University, Pattani, Thailand; 2 Department of Applied Life Science and Nanotechnology Research Center, Biomedical and Health Science College, Konkuk University, Chungju, Republic of Korea; 3 College of Oriental Medicine, Rangsit University, Pathumthani, Thailand; 4 Division of Hematology and Oncology, Icahn School of Medicine at Mount Sinai, New York, New York, United States of America; 5 Biomedical Technology Research Group for Vulnerable Populations and School of Health Science, Mae Fah Luang University, Muang, Chiang Rai, Thailand; University of Texas Medical Branch at Galveston, UNITED STATES OF AMERICA

## Abstract

Phy-Blica-O (PBO) is a traditional Thai polyherbal formulation historically regarded as a brain tonic and memory enhancer. Despite its long-standing ethnomedicinal use, its neuroprotective mechanisms have not been scientifically validated. This study provides the first experimental evidence that PBO alleviates lipopolysaccharide (LPS)-induced neuroinflammation (a process strongly linked to the development of neurodegenerative diseases) through the NF-κB signaling pathway. This study investigates the neuroprotective effects of PBO in lipopolysaccharide (LPS)-induced neuroinflammation, focusing on its ability to modulate the nuclear factor kappa B (NF-κB) signaling pathway *in vivo* and *in vitro*. The key constituents of PBO were quantified via high-performance liquid chromatography (HPLC). Antioxidant capacity was evaluated using DPPH, ABTS, and FRAP assays. The anti-neuroinflammatory effects of PBO were assessed in BV-2 microglial cells and male C57BL/6J mice challenged with LPS. Inflammatory mediators and cytokines were quantified at the mRNA and protein levels. NF-κB and MAPK signaling pathway activities were evaluated to elucidate the mechanisms of action of PBO. PBO pretreatment significantly reduced LPS-induced overproduction of nitric oxide (NO) (from 15.69 ± 1.63 to 8.74 ± 0.25 µM at 250 µg/mL, p < 0.001), pro-inflammatory cytokines (TNF-α, IL-1β, and IL-6 mRNA expression reduced by 25%, 31%, and 19%, respectively, p < 0.05), and inflammatory mediators (iNOS and COX-2 protein expression decreased by 41% and 29%, respectively, p < 0.05). Mechanistic analysis revealed that PBO exerts its protective effects primarily through inhibition of the NF-κB signaling pathway, reducing p-IκBα levels by 23% (p = 0.018) and p-p65 levels by 34% (p = 0.039) at 250 µg/mL in vitro, with no significant effect on MAPK signaling. These in vitro findings were corroborated by in vivo outcomes, where oral PBO administration (100 mg/kg for 7 days) significantly lowered iNOS and COX-2 mRNA expression (p = 0.041 and p = 0.018, respectively) and pro-inflammatory cytokine levels in the brains of LPS-challenged mice. Collectively, the results substantiate the traditional use of PBO as a neuroprotective tonic and highlight its potential as a cost-effective therapeutic candidate for preventing or managing neuroinflammatory conditions associated with neurodegeneration. Further studies are warranted to assess its bioavailability, long-term safety, and behavioral efficacy.

## Introduction

Neurodegenerative disorders are major causes of disability and morbidity worldwide and pose a growing public health concern due to their profound impact on aging populations [1,2]. Among these, Alzheimer’s disease (AD) is the most prevalent form of dementia, accounting for approximately 60%–80% of all cases [3]. The global prevalence of AD is projected to rise to 75 million by 2030 and exceed 132 million by 2050 [4]. In 2022, over 50 million individuals globally were affected by this neurocognitive disorder, with nearly 60% residing in low- and middle-income countries (LMICs), highlighting the extensive and growing burden of these conditions on global health systems [5]. Comparable to other LMICs, AD emerged as the predominant cause of dementia in Thailand, constituting 50–75% of diagnosed cases [6,7]. Despite reports indicating a dementia prevalence of approximately 2–3% within the Thai population [8,9], a recent finding has disclosed a significantly higher prevalence of mild cognitive impairment (MCI) among the Thai elderly, exceeding 70% [8].

The rising incidence of neurodegenerative disorders, coupled with the absence of targeted treatments for these conditions, increasing evidence supports the role of alternative therapies, including lifestyle modifications. Current pharmacological treatments provide only symptomatic relief and have a limited impact on disease progression. This gap has fueled interest in complementary approaches, particularly plant-based formulations, which are widely used in traditional medicine and often exhibit antioxidant and anti-inflammatory effects, thereby offering a promising and culturally relevant avenue for managing neurodegenerative diseases [10–14]. One notable example is Phy-Blica-O (PBO), also known as THP-R016, a traditional Thai polyherbal remedy used for decades as a brain tonic and memory enhancer. Our previous ethnopharmacological surveys in Southern Thailand identified PBO among 20 health tonics prescribed by folk healers, with its formulation attributed to Mr. Yop Lomsa of Songkhla province [15]. PBO contains 11 medicinal plants, including *Terminalia bellirica, Terminalia citrina,* and *Phyllanthus emblica*, all of which have reported antioxidant and anti-inflammatory properties. In experimental studies, PBO demonstrated strong antioxidant activity [15], improved stress resistance, and extended the lifespan of Caenorhabditis elegans under oxidative stress [16]. These findings support its ethnomedicinal reputation and highlight the need to explore its neuroprotective potential systematically.

Neuroinflammation is a critical driver of disease progression and a hallmark of neurodegenerative diseases. It is primarily mediated by activated microglia, which release pro-inflammatory cytokines such as interleukin-1β (IL-1β), tumor necrosis factor-α (TNF-α), and interleukin-6 (IL-6), and plays a decisive role in the progression of neurodegenerative disorders. Lipopolysaccharide (LPS)-induced neuroinflammation is widely used to model these processes in both in vitro and in vivo systems [17]. Mechanistically, LPS activates Toll-like receptor 4 (TLR4) on microglia, stimulating nuclear factor kappa B (NF-κB) and mitogen-activated protein kinase (MAPK) signaling cascades, which in turn amplify neuroinflammatory responses via the release of tumor necrosis factor-α (TNF-α), interleukin-1β (IL-1β), and interleukin-6 (IL-6). Against this backdrop, traditional formulations such as PBO may provide multi-target neuroprotection by modulating these pathways. Yet, despite its longstanding use in folk medicine, PBO’s role in neuroinflammation and its mechanistic underpinnings remain unexplored. This study investigates the neuroprotective mechanisms of PBO in LPS-induced neuroinflammation. Using a standardized decoction, we employed BV-2 microglial cells and mouse models to assess its effects, with a specific focus on NF-κB signaling. By combining chemical characterization, cellular assays, and in vivo validation, we aim to bridge traditional knowledge with modern neuropharmacology, thereby establishing scientific evidence for PBO as a potential neuroprotective formulation for neurodegenerative disorders.

## Materials and methods

### Preparation protocol of Phy-Blica-O decoction and determination of chemical contents

Phy-Blica-O (PBO) consists of equal parts from 11 medicinal plants, which are detailed in S1 Table, along with their taxonomic identities and voucher specimens. To replicate the traditional preparation method of PBO [15,16], 100 g of the dried herbal formula was boiled in 1000 mL of water at 96.3 ± 0.6 °C for 20 minutes. The resulting decoction was filtered and freeze-dried to yield a powdered extract. The percentage yield of the final freeze-dried powdered extract was 18.3 ± 1.2% (w/w), calculated as the mass of the dried extract relative to the initial dry weight of the herbal formula. For all assays, the extract was reconstituted in ethanol to a concentration of 25 mg/mL as the stock for all the experiments.

Representative chemical markers of PBO were identified through the qualitative analysis of its decoction [16] and selected based on their reported neuroprotective effects verified in both *in vitro* and animal studies [18–32]. Quantitative analysis was carried out using high-performance liquid chromatography (HPLC) [15,33]. Measurements were performed at a wavelength of 356 nm, and key chemical markers—ellagic acid, rutin, ferulic acid, chlorogenic acid, and caffeic acid—were counted by comparing their retention times and peak areas with those of authenticated reference standards. Batch-to-batch variability was evaluated, and results were expressed as milligrams per gram of dried extract.

### Determination of the antioxidant capacities of the PBO and its herbal constituents

The antioxidant capacities of PBO and its components were systematically quantified through a series of established assays, as reported in the literature [15,16,33]. These methodologies included the assessment of radical scavenging activities using 1,1-diphenyl-2-picrylhydrazyl (DPPH) and 2,2’-azino-bis (3-ethylbenzothiazoline-6-sulfonic acid) (ABTS), ferric reducing antioxidant power (FRAP), superoxide anion radical scavenging, and peroxyl radical scavenging capacity. For each assay, PBO was tested in triplicate at concentrations ranging from 25 to 500 μg/mL. IC_50_ values were calculated to compare the antioxidant capacity of PBO with its individual herbal components.

### Effect of the PBO administration on the survival of cells and the production of NO in BV2 cells stimulated with LPS

BV-2 microglial cells were maintained in Dulbecco’s Modified Eagle Medium (DMEM) sustained with 10% fetal bovine serum and 1% penicillin–streptomycin. Cells were planted into six-well plates at a density of 5 × 10^5^ cells per well, and inflammatory responses were induced by treatment with 200 ng/mL lipopolysaccharide (LPS) derived from *Escherichia coli* (O111:B4). To mimic the neuroprotective effect of PBO and the inflammatory environment in the brain, PBO was added at concentrations of 50, 125, and 250 μg/mL one hour before LPS stimulation [34].

Cell viability was assessed using the MTT assay. Following treatments, 20 μL of MTT solution (5 mg/mL) was added to each well and incubated for 4 hours. Formazan crystals were dissolved in 100 μL of dimethyl sulfoxide (DMSO), and absorbance was calculated at 570 nm using a microplate reader. Nitric oxide (NO) production was determined using the Griess reagent. Culture supernatants (50 μL) were incubated for 15 minutes after being mixed with an equal volume of Griess reagent. Absorbance was measured at 540 nm, and a sodium nitrite standard curve was used to estimate the nitrite concentrations.

### Gene expression analysis by reverse transcriptase polymerase chain reactions (RT-PCR)

Total RNA was extracted from BV-2 cells using the Trizol reagent (Invitrogen), and cDNA synthesis was accomplished with the ReverTra Ace qPCR RT Master Mix (Toyobo, Osaka, Japan). The mRNA expression levels of interleukin-6 (IL-6), tumor necrosis factor-α (TNF-α), cyclooxygenase-2 (COX-2), inducible nitric oxide synthase (iNOS), and interleukin-1β (IL-1β) were calculated by reverse transcriptase polymerase chain reaction (RT-PCR). GAPDH was used as an internal control. Band intensities were quantitatively assessed using densitometry analysis, employing the multi-gauge software V3.1 (Fujifilm, Tokyo, Japan) [34].

### Western blotting analysis

Protein expression levels of iNOS, COX-2, NF-κB/p65, and IκBα were analyzed by Western blotting. Lysates were prepared using RIPA buffer, and protein concentrations were measured using the Bradford assay. Proteins with equal amounts were separated by 10% sodium dodecyl sulfate-polyacrylamide gel electrophoresis, followed by determination of proteins that were transferred to polyvinylidene difluoride membranes (Millipore, Bedford, MA, USA) in complying with earlier published protocols [35,36]. Membranes were blocked by non-fat milk at 5% and incubated with primary antibodies specific to target proteins overnight. Protein expression was quantified with ImageJ software after being visualized through an enhanced chemiluminescence detection system (Santa Cruz Biotechnology).

### In vivo protective mechanisms of PBO on LPS-induced neuroinflammation

The animal experiments strictly adhered to the guidelines of the Institutional Animal Care and Use Committee (IACUC) of Konkuk University (Approval No. KUB19520). All procedures were approved by the IACUC of Konkuk University, Republic of Korea. No additional field site access permits were required. All experiments were conducted entirely under controlled laboratory conditions at Konkuk University. Male C57BL/6J mice (8–9 weeks old, 24–27 g) were used in this study. Only male mice were used to minimize hormonal variability that could confound neuroinflammatory responses; future studies should include both sexes to assess potential sex-dependent effects. Animals were kept under controlled atmosphere with a regulated temperature and a 12/12-hour dark/light cycle. All animals had free access to food and water and underwent a 7-day acclimatization period prior to the study

A total of 42 mice were randomly assigned to seven groups (n = 6 per group), as summarized in Table 1. Briefly, one group served as control, two groups received distilled water prior to LPS injection, and four groups were pretreated with PBO (50 or 100 mg/kg) before LPS injection. Animals were observed for general activity, grooming, posture, respiration and food intake. Humane endpoints were predefined and included persistent weight loss greater than 15%, severe lethargy, impaired mobility, respiratory distress, inability to access food or water or any condition judged by the attending veterinarian as requiring intervention. No animals reached humane endpoints during the study, and no deaths occurred before scheduled euthanasia. At the end of the exposure period, sacrifice was performed at either 3- or 6-hour post-LPS after animals were subsequently euthanized under deep anesthesia using intraperitoneal ketamine–xylazine (100 mg ketamine/kg and 10 mg xylazine/kg) cocktail intraperitoneally, in accordance with AVMA-approved methods. No unplanned deaths occurred, and survival was not used as an experimental endpoint. All efforts were made to minimize suffering. The experimental workflow is shown in Fig 6A. Whole brains were collected for RT-PCR and Western blot analyses [34,36,37].

**Table 1:** Experimental groups and treatments.

Group (n = 6)	Pretreatment (7 days)	LP (3 mg/kg i.p.)	Sacrifice time
1	None (Control)	None	–
2	Distilled water	Yes	3h
3	Distilled water	Yes	6h
4	PBO (50 mg/kg)	Yes	3h
5	PBO (50 mg/kg)	Yes	6h
6	PBO (100 mg/kg)	Yes	3h
7	PBO (100 mg/kg)	Yes	6h

**Fig 1:**
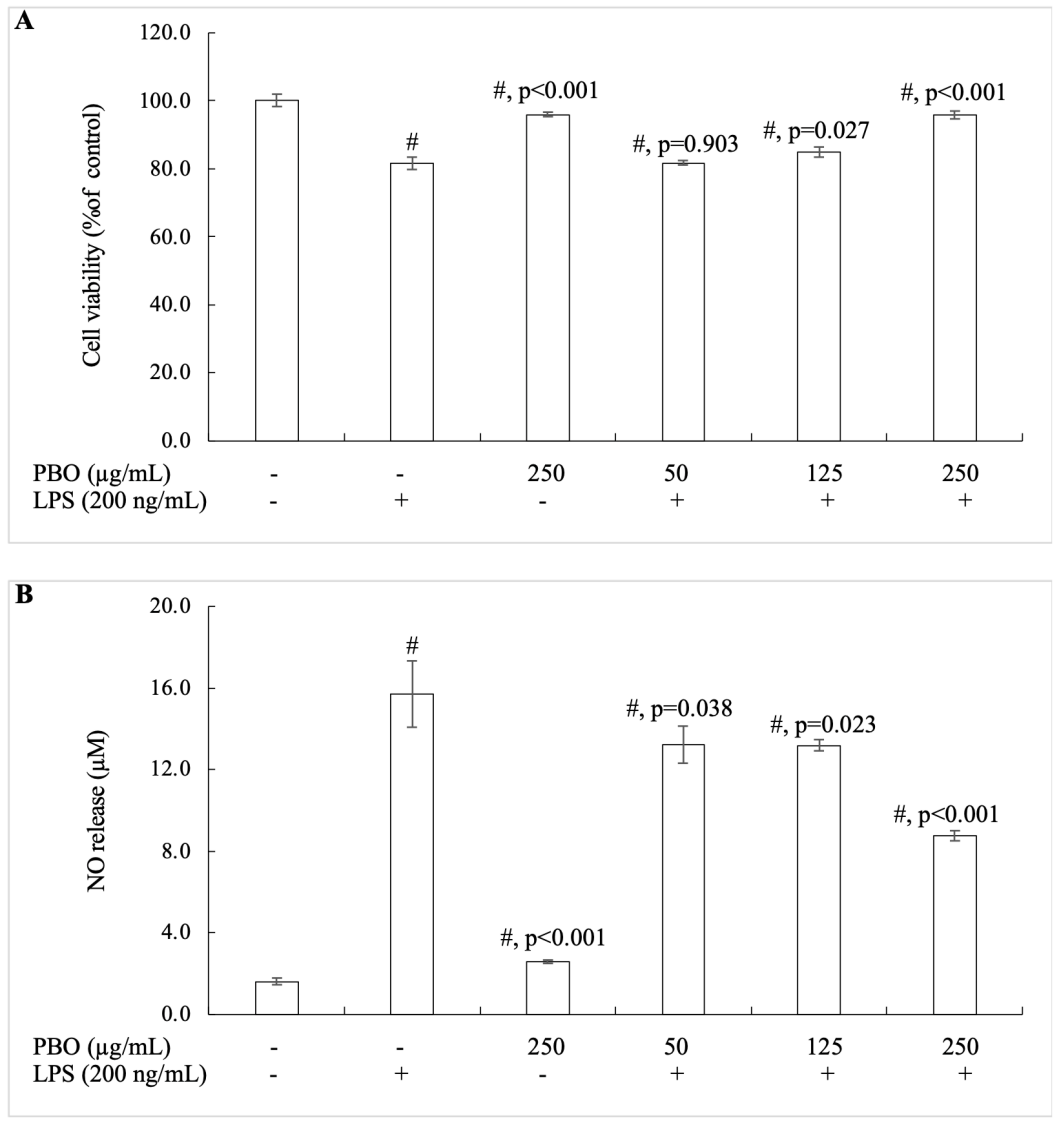
The dose-response effects of the Phy-Blica-O (PBO) decoction on BV-2 microglial cell viability (A) and its influence on nitric oxide (NO) production induced by lipopolysaccharide (LPS) in BV-2 cells (B). BV-2 cells were pretreated with different concentrations (50-250 µg/mL) of PBO for one hour, after which the cells were treated with LPS (200 ng/mL) for 24 h. Cell viability and nitric oxide production percentage were expressed as mean±SEM (n = 3). Each p-value (p < 0.05) represents the statistical difference compared with the LPS-treated group, whereas ‘#’ denotes p < 0.05 compared with the control group.

**Fig 2:**
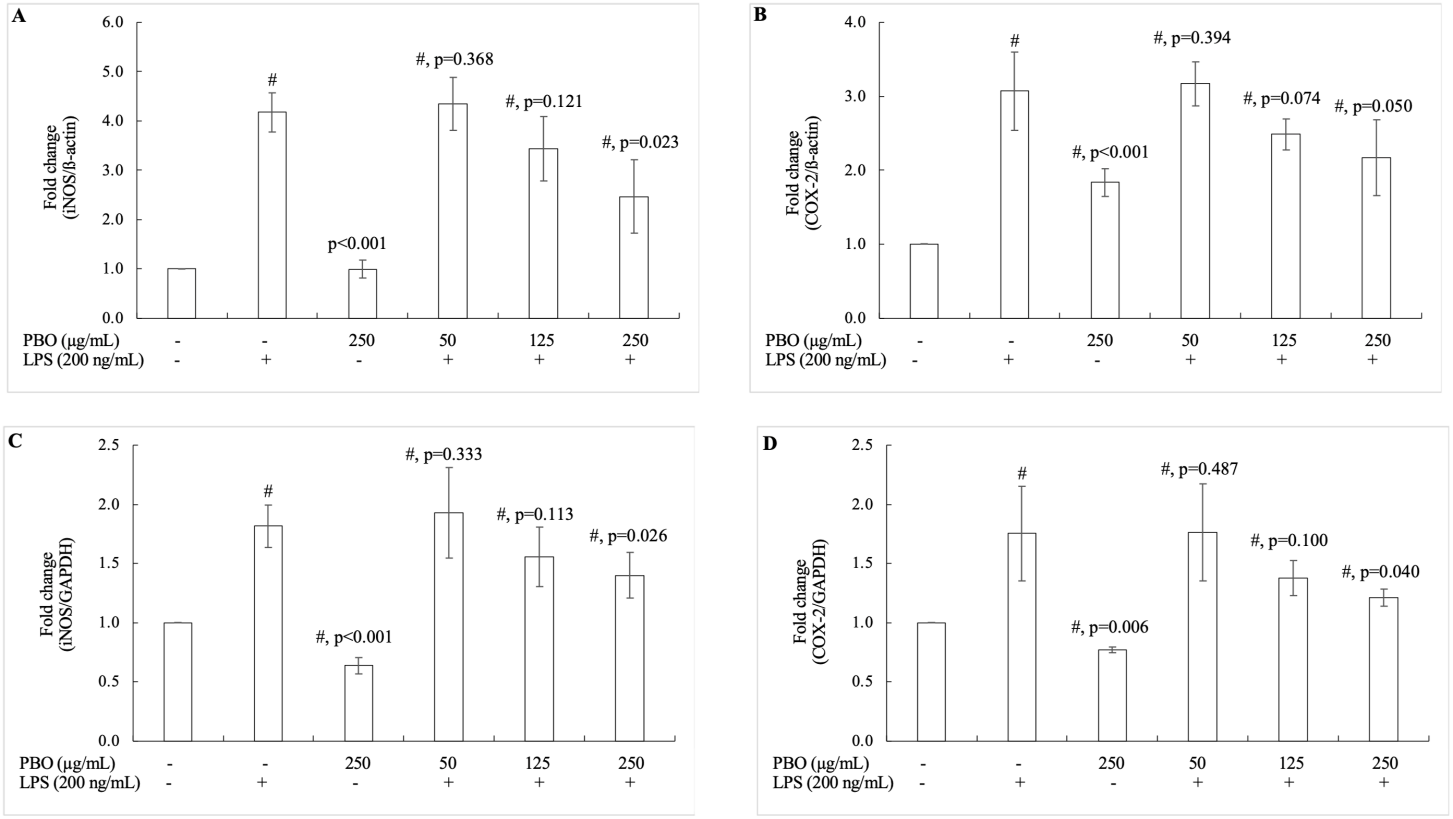
Phy-Blica-O (PBO) decoction attenuated the iNOS (A) and COX-2 production (B) in LPS-stimulated BV-2 microglial cells. Fold gene expression changes are also shown for iNOS (**C**) and COX-2 (**D**). The data are presented as means±SEM (n = 3). Each p-value (p < 0.05) represents the statistical difference compared with the LPS-treated group, whereas ‘#’ denotes p < 0.05 compared with the control group.

**Fig 3:**
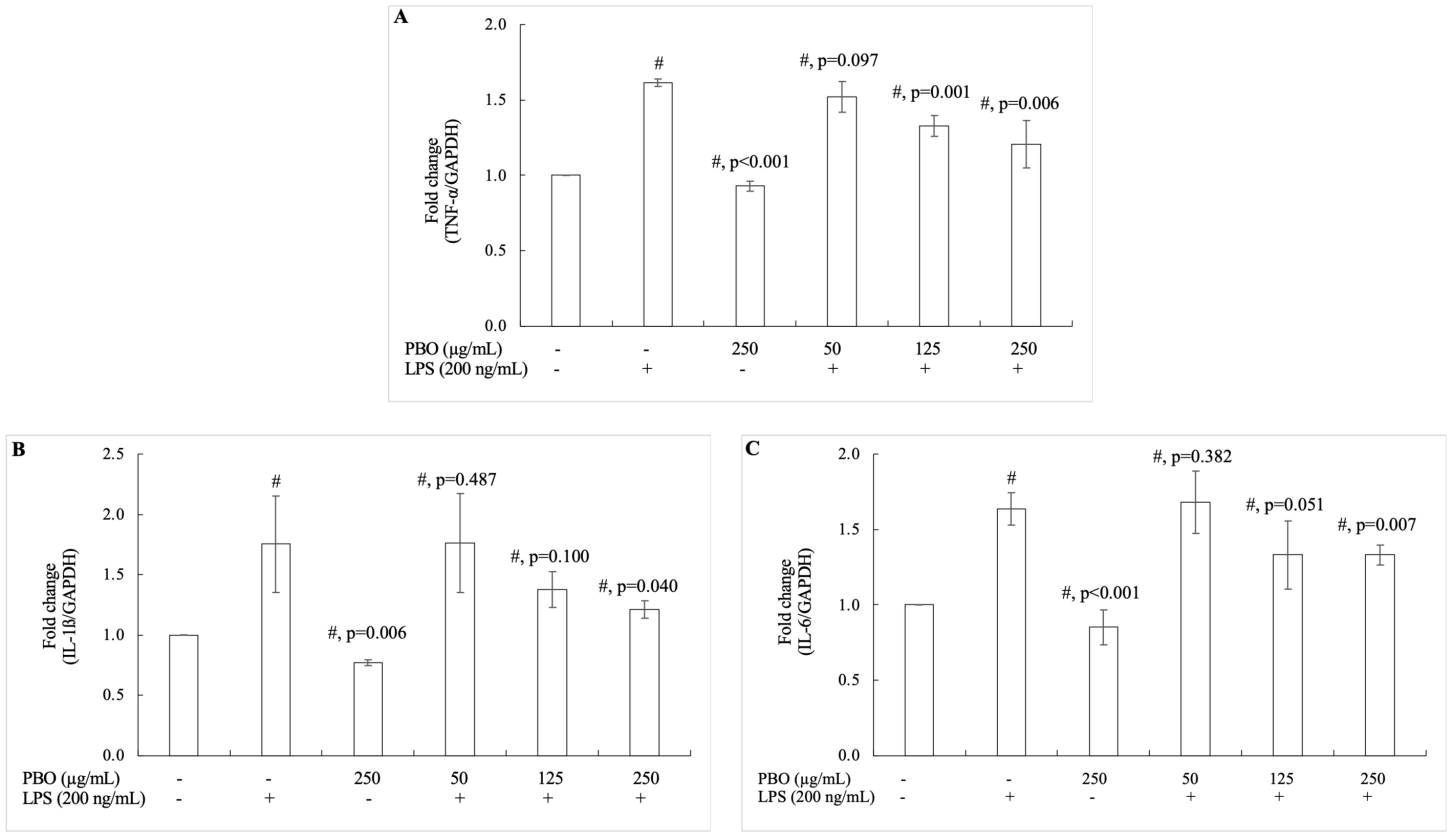
Phy-Blica-O (PBO) decoction attenuated gene expression of pro-inflammatory cytokines in the LPS-stimulated BV-2 microglial cells. Fold-changes in gene expression are presented for tumor necrosis factor-α (TNF-α; **A)**, interleukin-1β (IL-1β; **B)**, and IL-6 (C) measured with real-time PCR analysis. The data are presented as means±SEM (n = 3). Each p-value (p < 0.05) represents the statistical difference compared with the LPS-treated group, whereas ‘#’ denotes p < 0.05 compared with the control group.

**Fig 4:**
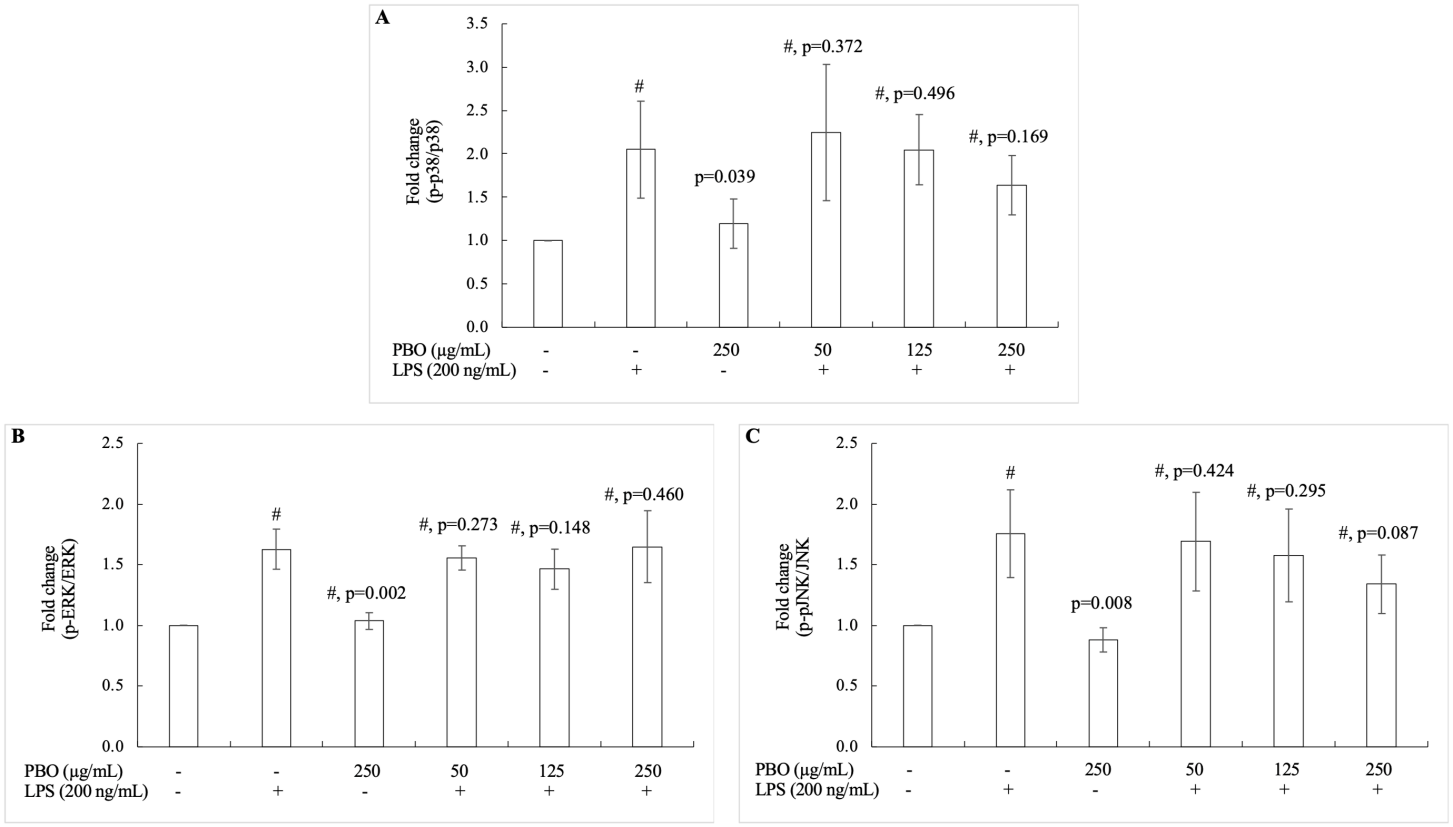
Effects of pretreatment with Phy-Blica-O (PBO) decoction on mitogen-activated protein kinases (MAPKs) pathway in LPS-stimulated BV-2 microglial cells. Fold changes in protein production of p-p38/p38 **(A)**, p-ERK/ERK **(B)**, and p-JNK/JNK (C) are presented as mean±SEM (n = 3). Each p-value (p < 0.05) represents the statistical difference compared with the LPS-treated group, whereas ‘#’ denotes p < 0.05 compared with the control group.

**Fig 5:**
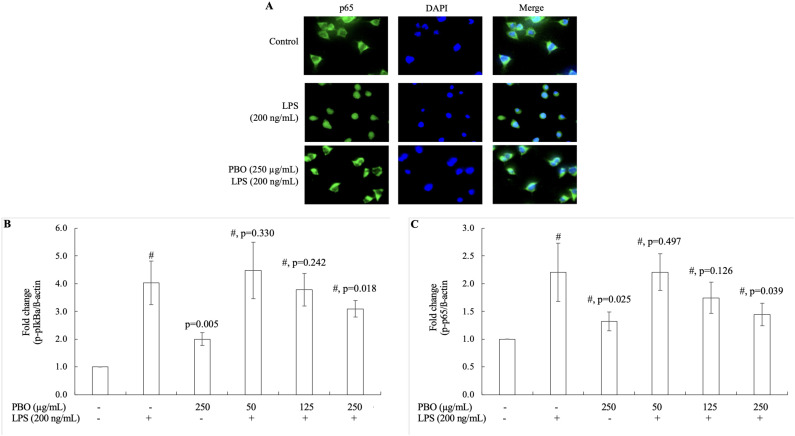
Effects of pretreatment with Phy-Blica-O (PBO) decoction on protein expression levels of NF-κB measured in LPS-induced BV-2 microglial cells. Immunofluorescence assay determined representative images of NF-κB (p65) localization in LPS-stimulated BV-2 microglial cells and compared with BV-2 control cells **(A)**. Effects of PBO decoction on p-IκBα (B) and p-p65 (C) protein expression levels in LPS-induced BV-2 microglial cells. The data are presented as mean±SEM (n = 3). Each p-value (p < 0.05) represents the statistical difference compared with the LPS-treated group, whereas ‘#’ denotes p < 0.05 compared with the control group.

### Statistic

All results are expressed as mean±SEM or mean±SD. Comparisons between the experimental and vehicle groups were achieved via one-way analysis of variance (ANOVA) with SPSS software Version 19.0 (IBM SPSS, Inc., Chicago, IL, http://www.ibm.com). A p-value of ≤ 0.05 was thought statistically significant.

## Results and discussion

### Ellagic acid, rutin, ferulic acid, and chlorogenic acid are neuroprotective marker compounds in Phy-Blica-O decoction (PBO)

The chemical characterization of PBO identified ellagic acid, rutin, ferulic acid, and chlorogenic acid as key neuroprotective markers (**Table 2**). Ellagic acid was the most abundant compound, measured at 29.734 mg/g, significantly exceeding the concentrations of the other markers. It was found to be significantly higher than the concentration of chlorogenic and ferulic acids and about 600-fold that of caffeic acid. Rutin followed at 16.335 mg/g, while chlorogenic and ferulic acids were present in similar amounts (~0.46 mg/g). These findings establish a baseline for the standardization of PBO and its potential neuroprotective capacity.

**Table 2:** Proposed neuroprotective marker compounds measured from the Phy-Blica-O (PBO) decoction. Reported neuroprotective effects are supported by key references as cited in the table.

Test constituents (RT, min)	Contents (mg/g)	Reported neuroprotective effects	
		*In vitro*	*In vivo*
Chlorogenic acid (1.609)	0.462 ± 0.034	LPS-stimulated BV2 microglial cells	MPTP-mediated apoptotic cascade in mice [29,32]
Caffeic acid (2.563)	0.046 ± 0.005	H_2_O_2_ -treated PC12 cells	An Aβ25–35-induced Alzheimer’s disease in mice, MPTP-treated. [20,24]
Ferulic acid (5.241)	0.464 ± 0.006	BaP-induced BV2 microglial cells. MPP + /MPTP-challenged SH-SY5Y cells	Aβ-injected mice, rotenone-induced rat MPTP-treated mice [19,25,27]
Rutin (3.835)	16.335 ± 0.036	SGD-induced PC12 cells Amylin-induced -induced SH-SY5Y cells	Aβ-injected rats, 6-OHDA Parkinson’s disease in rats [23,26,28,31]
Ellagic acid (4.41)	29.734 ± 0.285	LPS-stimulated BV2 microglial cells	MPTP-treated mice LPS-induced DA neuronal damage in rats [18,21,22,38]

To further confirm the identity of these marker compounds, an HPLC fingerprint analysis of PBO was performed and compared against authenticated reference standards. The chromatographic profiles of PBO showed peaks at retention times corresponding to those of chlorogenic acid (1.609 min), caffeic acid (2.563 min), rutin (3.835 min), ellagic acid (4.41 min), and ferulic acid (5.241 min), consistent with the reference standards (see Table 2). The co-elution of PBO peaks with standard compounds at identical retention times, together with comparable UV absorption spectra at 356 nm, confirmed the presence and identity of each constituent. This fingerprint approach provides a reliable and reproducible quality control measure for PBO batch standardization.

### *Terminalia citrina, Terminalia bellirica, Terminalia arjuna*, and *Phyllanthus emblica* contribute significantly to the antioxidant properties of PBO

As shown in Table 3, *in vitro* antioxidant assays revealed strong free radical scavenging abilities of *T. citrina*, *T. bellirica*, *T. arjuna*, and *P. emblica*, which are key constituents of PBO. This is evidenced by their lower IC_50_ values in the DPPH, ABTS, and NBT assays and higher values in the FRAP assay, approximately 2–3 times greater than those of the PBO extract. Additionally, the extracts from *T. citrina* and *T. arjuna* show more potent peroxyl radical scavenging activity than the PBO extract, as indicated by the ORAC assay. These components exhibited superior activity in mixed-mode, electron transfer-based, and hydrogen atom transfer-based assays compared to the complete PBO decoction, highlighting their individual contributions to the overall antioxidant capacity of this formulation.

**Table 3:** Comparison of the free radical scavenging properties of Phy-Blica-O (PBO) decoction and its herbal components.

Tested Extracts	MA (IC50; µg/mL) *	ETBA		HATA	
	DPPH	ABTS	FRAP (mM Fe_2_SO_4_/mg sample)	NBT (IC_50_) (mg/mL)	ORAC (mM of TE/µg extract)
Phy-Blica-O	16.52 ± 0.31	82.58 ± 1.31	5.95 ± 0.07	0.21 ± 0.00	5.41 ± 0.51
*Allium sativum*	1758.35 ± 80.67	269.29 ± 13.39	0.48 ± 0.00	1.91 ± 0.12	0.46 ± 0.03
*Alpinia galanga*	175.42 ± 7.38	267.15 ± 1.42	0.85 ± 0.02	1.31 ± 0.08	4.91 ± 0.09
*Cyperus rotundus*	177.02 ± 10.87	652.43 ± 10.57	1.07 ± 0.01	8.11 ± 0.48	1.41 ± 0.05
*Maerua siamensis*	117.34 ± 2.45	251.95 ± 6.99	1.77 ± 0.01	0.24 ± 0.01	2.71 ± 0.18
*Phyllanthus emblica*	8.27 ± 0.18	43.40 ± 0.83	10.06 ± 0.10	0.07 ± 0.00	5.39 ± 0.34
*Piper retrofractum*	618.30 ± 42.43	1155.79 ± 9.00	0.80 ± 0.02	0.72 ± 0.05	0.99 ± 0.01
*Terminalia arjuna*	3.61 ± 0.07	18.11 ± 0.53	13.79 ± 0.30	0.05 ± 0.00	8.79 ± 0.40
*Terminalia bellerica*	4.94 ± 0.06	36.50 ± 0.54	12.39 ± 0.07	0.05 ± 0.00	4.27 ± 0.11
*Terminalia citrine*	4.64 ± 0.08	29.06 ± 1.41	13.33 ± 0.50	0.05 ± 0.00	9.35 ± 0.17
*Tinospora crispa*	128.06 ± 1.24	206.58 ± 4.77	1.66 ± 0.01	0.82 ± 0.03	3.52 ± 0.21
*Zingiber officinale*	136.34 ± 4.54	676.07 ± 17.21	0.84 ± 0.02	3.33 ± 2.11	1.05 ± 0.03

MA (Mixed-mode assays); ETBA (Electron transfer-based assays); HATA (Hydrogen atom transfer-based assay) *Trolox was added as a positive control for DPPH and ABTS assays; its IC_50_ was found to be 69.71 ± 1.04 and 1166.26 ± 2.87 µg/mL, respectively. ^#^Catechin was added as a positive control for the NBT assay; its IC_50_ was 0.008 ± 0.000 mg/mLPBO pretreatment mitigates LPS-induced cytotoxicity and NO overproduction in BV-2 cells.

Fig 1 illustrates the dose-response effects of the PBO decoction on BV-2 microglial cell viability and its impact on NO production induced by LPS in BV-2 cells. Pretreatment with PBO demonstrated a dose-dependent protective effect against LPS-induced cytotoxicity.

Pretreatment with this decoction for one hour appears to confer protection against LPS-induced cytotoxicity in a dose-dependent manner, with a marked increase in cell viability in comparison to the LPS-only treatment, showing cell viability percentages of 81.69 ± 0.72 (p = 0.903), 84.93 ± 1.45 (p = 0.027), and 95.85 ± 1.19 (p < 0.001) at the PBO concentrations of 50, 125, and 250 µg/mL, respectively.

Furthermore, pretreating with the PBO at increasing concentrations leads to a dose-dependent reduction in LPS-induced NO production. The greatest concentration of PBO (250 µg/mL) results in the most substantial decrease in NO production, dropping from 15.69 ± 1.63 µM in the non-pretreated sample to 8.74 ± 0.25 µM in the PBO-pretreated group, indicating that this decoction exerts a significant inhibitory effect on LPS-induced NO production.

It is noteworthy that in several in vitro assays, PBO-alone-treated groups (in the absence of LPS) exhibited statistically significant differences compared with the untreated control group. This observation is likely attributable to the intrinsic bioactivity of the polyphenolic constituents of PBO, particularly its abundance of ellagic acid (29.734 mg/g) and rutin (16.335 mg/g), which possess direct antioxidant and immunomodulatory properties. At higher concentrations (125–250 µg/mL), these compounds may exert mild baseline suppression of constitutive inflammatory mediator production in non-stimulated BV-2 cells, as has been reported for polyphenol-rich extracts in unstimulated microglia. These effects were modest in magnitude and did not compromise cell viability, indicating that PBO exerts tonic anti-inflammatory activity even in the absence of an exogenous inflammatory stimulus.

### PBO suppresses LPS-induced pro-inflammatory mediators and cytokines in BV-2 cells

Pro-inflammatory mediators and cytokines, including iNOS, COX-2, tumor necrosis factor α (TNF-α), interleukin 1β (IL-1β), and IL-6, are essential in regulating the inflammatory response. To assess whether the PBO decoction influences iNOS and COX-2 production, the levels were measured at both the transcriptional and translational levels in LPS-stimulated BV-2 microglial cells. The data indicate that PBO exerts a dose-dependent inhibitory effect on both mRNA and protein expression levels of iNOS and COX-2 in microglial cells following LPS stimulation (**Fig 2**). PBO pretreatment significantly attenuated the fold change in mRNA and protein expression of iNOS upon LPS stimulation, contrasted to cells that were not pretreated. The fold changes were 4.173 ± 0.399 vs. 2.467 ± 0.738 (p = 0.023) for protein expression and 1.814 ± 0.181 vs. 1.399 ± 0.192 (p = 0.026) for mRNA expression. Similarly, this pretreatment also affected the fold changes in mRNA and protein expressions of COX-2 compared to the unpretreated cells. The fold changes in mRNA and protein expressions of COX-2 in the pretreated cells following LPS stimulation were 1.208 ± 0.073 and 2.167 ± 0.417, respectively, which were lower than those in the unpretreated cells, where the fold changes were 1.752 ± 0.398 for mRNA expression (p = 0.040) and 3.068 ± 0.429 for protein expression (p = 0.050).

Furthermore, PBO pretreatment significantly attenuated the expression of TNF-α, IL-1β, and IL-6 in LPS-stimulated BV-2 cells, demonstrating its potent anti-neuroinflammatory effects (Fig 3). Pretreatment with the PBO decoction suppressed the secretion of these cytokines in a dose-dependent manner and significantly attenuated their mRNA expression in response to LPS stimulation at a concentration of 250 µg/mL. Specifically, TNF-α expression was reduced from 1.613 ± 0.025 post-LPS treatment to 1.327 ± 0.069 (p = 0.001) and 1.206 ± 0.158 (p = 0.006) when cells were pretreated with the decoction at concentrations of 125 and 250 µg/mL, respectively. Similar reductions were observed in the secretion of IL-1β and IL-6. The fold changes in IL-1β and IL-6 expression were significantly diminished by PBO treatment (250 µg/mL) from 1.752 ± 0.398 to 1.208 ± 0.073 (p = 0.040) and from 1.638 ± 0.108 to 1.330 ± 0.065 (p = 0.007), respectively.

### PBO modulates the activation of nuclear factor kappa B (NF-κB) but not the mitogen-activated protein kinase (MAPK) signaling pathway in BV-2 cells

The expression of pro-inflammatory molecules is controlled through the inducible activation of genes, which is mediated by the stimulation of MAPK and NF-κB signaling pathways [39]. Mechanistic analysis revealed that PBO inhibited NF-κB activation by reducing the phosphorylation of IκBα and p65. At 250 μg/mL, p-IκBα levels decreased by 23% (p = 0.018), and p-p65 levels by 34% (p = 0.039; Fig 4). Conversely, PBO had minimal effects on the phosphorylation of MAPK proteins (ERK, JNK, and p38; Fig 5), indicating specificity in targeting the NF-κB pathway.

### PBO attenuates LPS-induced neuroinflammation in vivo

**Fig 6:**
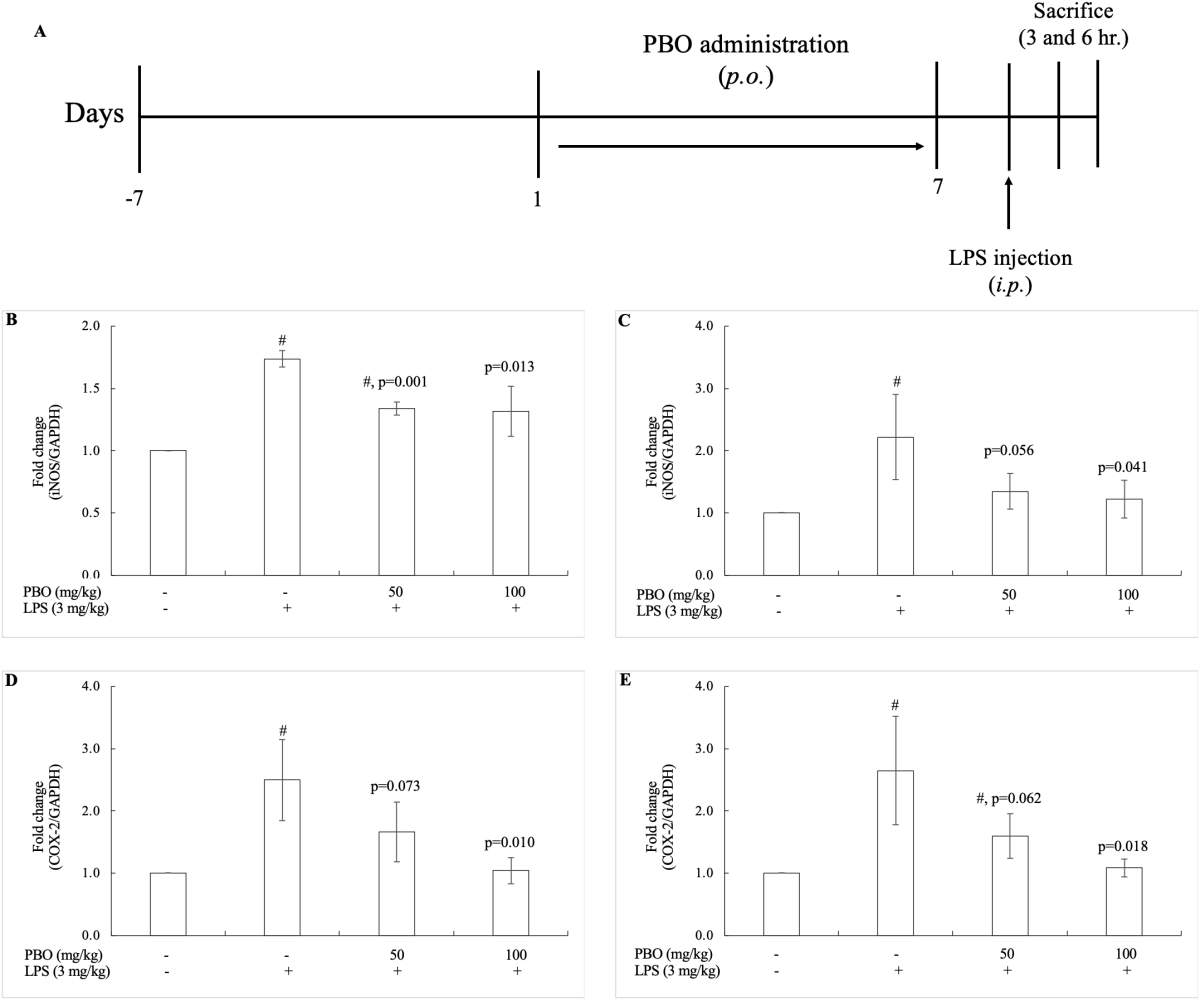
An experimental procedure was performed to evaluate the effect of Phy-Blica-O (PBO) decoction on LPS-induced neuroinflammation in the mouse brain. **(A)** Mice (n = 6 per group) were given PBO at 50 and 100 mg/kg, once daily for seven consecutive days, and injected with LPS at 3 mg/kg on the 8^th^ day. Inhibitory effects of LPS-mediated iNOS (B and C) and COX-2 (D and E) by PBO were analyzed at 3 hr (B and D) and 6 hr (C and E) after injection. The data are presented as mean±SEM (n = 6 per group; mRNA analyses used n = 3 randomly selected animals per group). Each p-value (p < 0.05) represents the statistical difference compared with the LPS-treated group, whereas ‘#’ denotes p < 0.05 compared with the control group.

The preventive potential of PBO administration in suppressing neuroinflammation was investigated using an LPS-induced mouse model. Consuming the PBO decoction for seven consecutive days at a dosage of 100 mg/kg significantly prevented LPS-induced neuroinflammation, as indicated by the suppressed mRNA and protein expression levels of iNOS and COX-2. The mRNA expression levels of iNOS and COX-2 decreased from 2.218 ± 0.685 to 1.221 ± 0.301 (p = 0.041) and 2.648 ± 0.869 to 1.083 ± 0.139 (p = 0.018), respectively, as shown in Fig 6.

**Fig 7:**
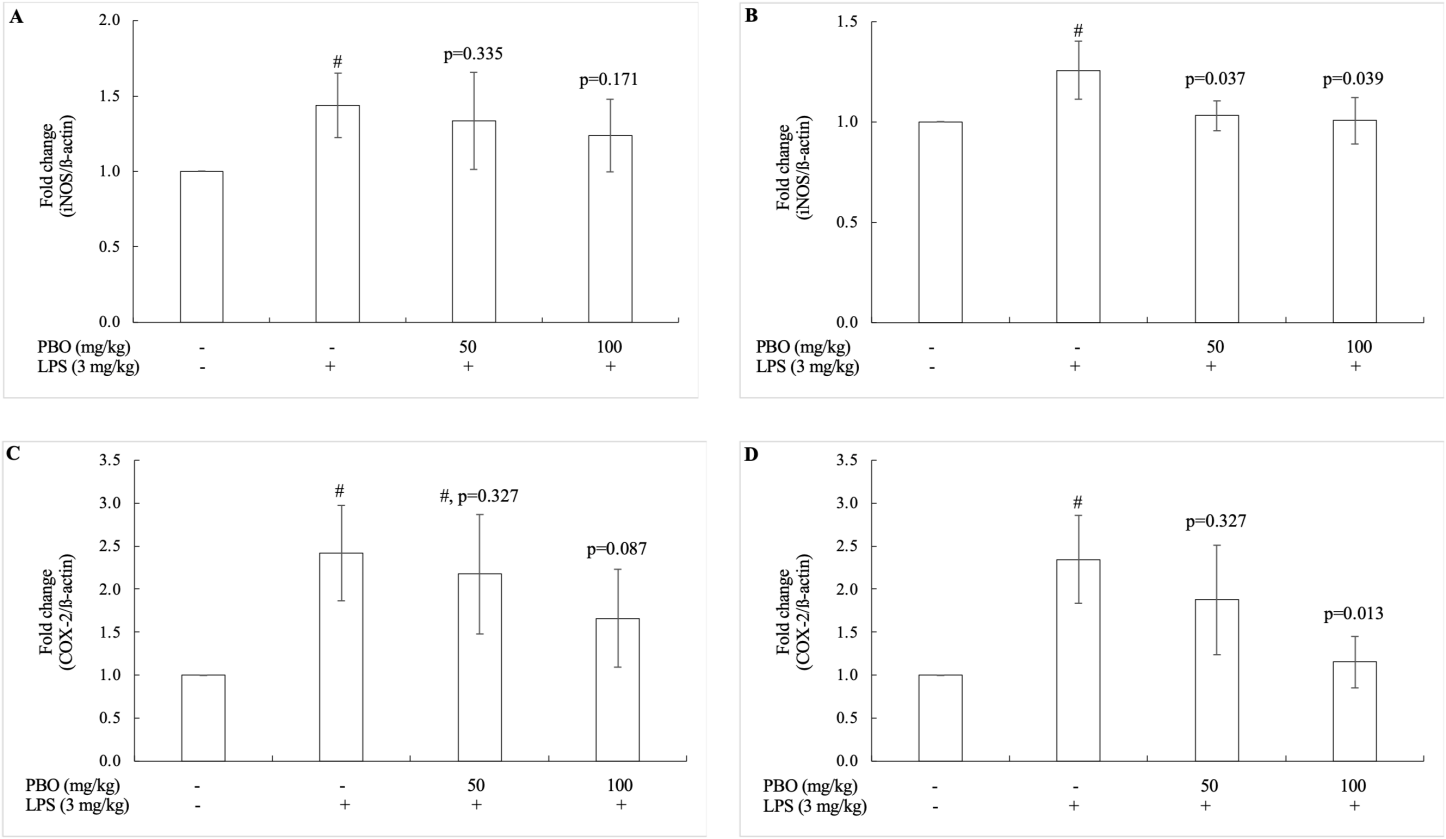
Inhibitory effects of Phy-Blica-O (PBO) decoction towards iNOS (A and B) and COX-2 (C and D) protein expression levels in brain homogenates obtained from LPS-mediated neuroinflammation mice at 3 hr (A and C) and 6 hr (B and D) after LPS (3 mg/kg) injection. The relative amounts of iNOS and COX-2 were normalized with ß-actin using a densitometer. The data were presented as mean±SEM (n = 6 per group; protein analyses used n = 3 randomly selected animals per group). Each p-value (p < 0.05) represents the statistical difference compared with the LPS-treated group, whereas ‘#’ denotes p < 0.05 compared with the control group.

Similarly, the fold changes in protein production of these pro-inflammatory mediators, as observed through western blot analysis, significantly declined from 1.258 ± 0.145 to 1.006 ± 0.116 (p = 0.039) for iNOS and from 2.346 ± 0.515 to 1.151 ± 0.296 (p = 0.013) for COX-2, as depicted in Fig 7.

**Fig 8:**
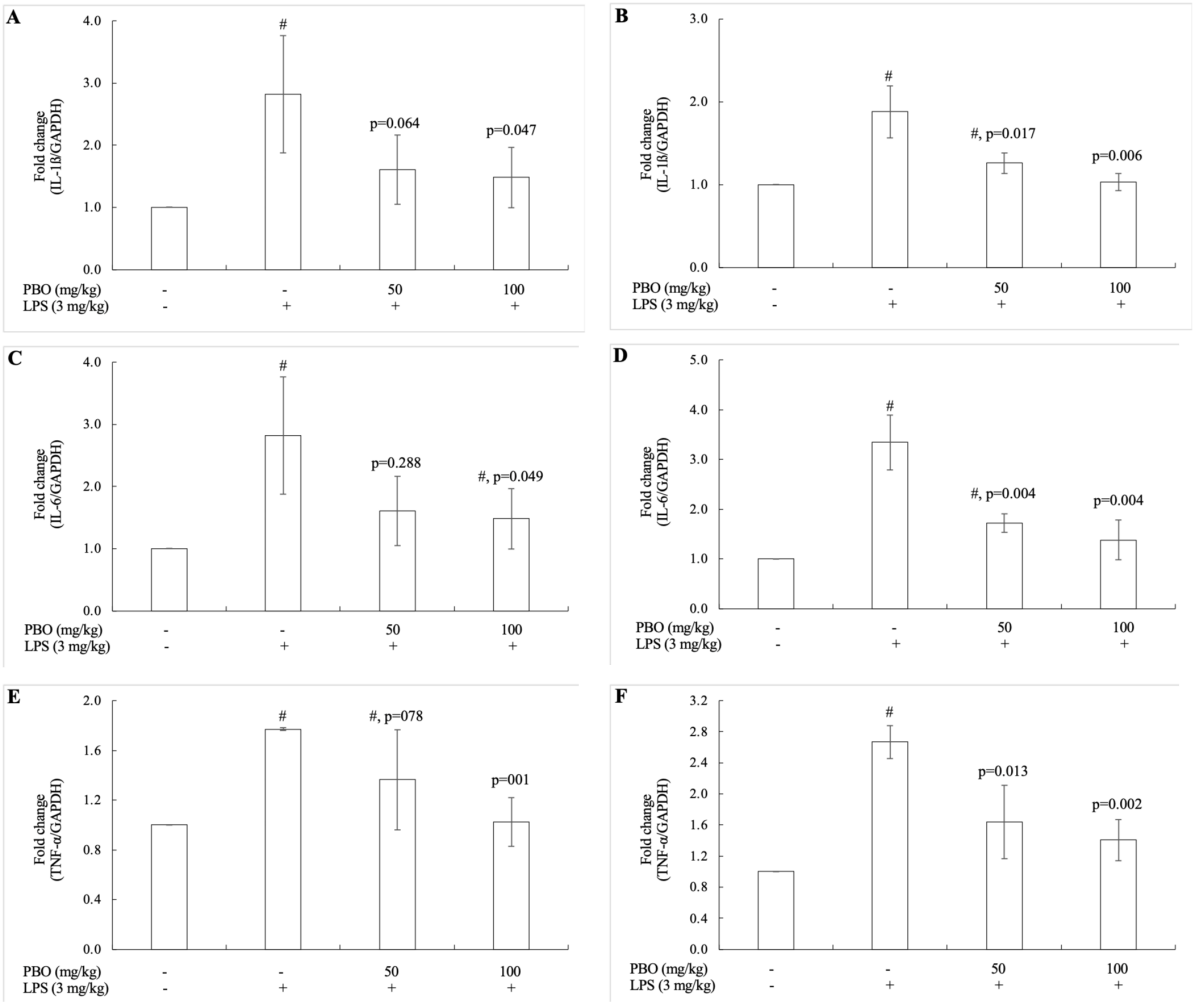
Inhibitory effect of Phy-Blica-O (PBO) decoction towards pro-inflammatory cytokines including interleukin (IL)-1ß (A and B), IL-6 (C and D) and tumor necrosis factor (TNF)-α (E and F) mRNA expression levels in brain homogenates obtained from LPS-mediated neuroinflammation mice at 3 hr (A, C, and E) and 6 hr (B, D, and F) after LPS (3 mg/kg) injection. The relative amounts of IL-1ß, TNF-α, and IL-6 mRNA were normalized with GAPDH mRNA. The data are presented as means±SEM (n = 6 per group; mRNA analyses used n = 3 randomly selected animals per group). Each p-value (p < 0.05) represents the statistical difference compared with the LPS-treated group, whereas ‘#’ denotes p < 0.05 compared with the control group.

Pro-inflammatory cytokines TNF-α, IL-1β, and IL-6 also exhibited marked reductions in mRNA levels (Fig 8). The fold changes in mRNA expression of TNF-α, IL-1β, and IL-6 at six hours post-LPS injection in the PBO-treated group were 1.407 ± 0.264 (p = 0.002), 1.032 ± 0.107 (p = 0.006), and 1.379 ± 0.396 (p = 0.004), respectively. These levels were significantly lower compared to the LPS-induced untreated group, which showed expressions of 2.667 ± 0.214, 1.032 ± 0.107, and 3.343 ± 0.551 for TNF-α, IL-1β, and IL-6, respectively.

**Fig 9:**
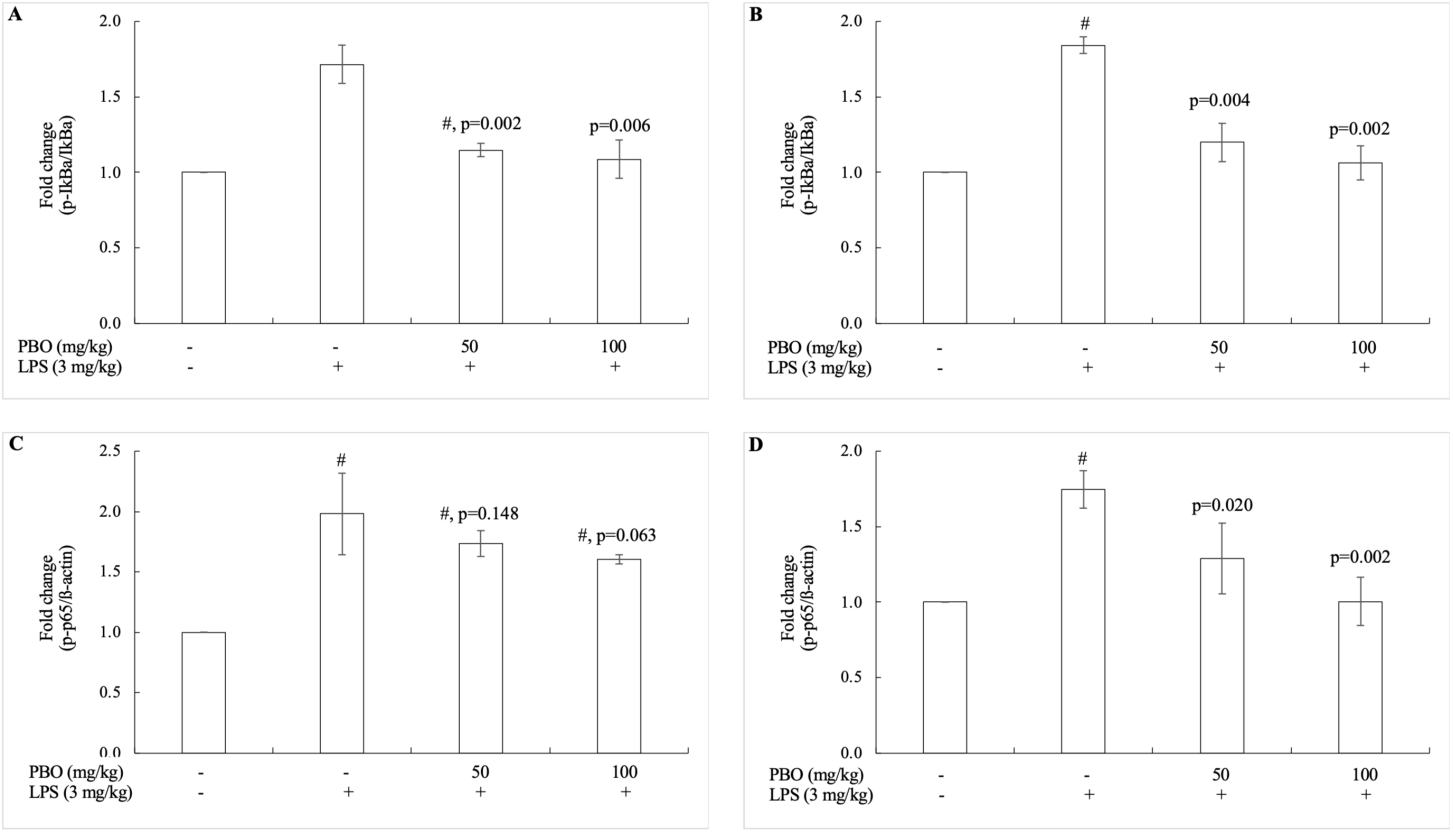
Inhibitory effects of Phy-Blica-O (PBO) decoction on protein expression levels of phosphorylated IκBα (p-IκBα; A and B) and phosphorylated p65 (p-p65; C and D) in brain homogenates obtained from LPS-mediated neuroinflammation mice at 3 hr (A and C) and 6 h (B and D) after LPS (3 mg/kg) injection. A densitometer normalized the relative amounts of p-IκBα and p-p65 with ß-actin. The data is presented as means±SEM (n = 6 per group; protein analyses used n = 3 randomly selected animals per group). Each p-value (p < 0.05) represents the statistical difference compared with the LPS-treated group, whereas ‘#’ denotes p < 0.05 compared with the control group.

The results further confirm that PBO supplementation prevents LPS-induced neuroinflammation by inactivating the NF-κB signaling pathway, as evidenced by the dramatic reduction in the phosphorylation of IκBα and p65 in the brain tissue (Fig 9). Six hours post-LPS injection, the fold changes in p-IκBα for groups supplemented with 50 and 100 mg/kg of PBO were 1.198 ± 0.125 (p = 0.004) and 1.064 ± 0.113 (p = 0.002), respectively, compared to 1.842 ± 0.054 in the untreated group.

Similarly, p-p65 protein expression in the PBO-supplemented group significantly decreased in a dose-dependent manner, from 1.745 ± 0.123 in the untreated brain to 1.289 ± 0.234 (p = 0.020) and 1.003 ± 0.159 (p = 0.002) at 50 and 100 mg/kg, respectively.

The collective results from *in vitro* and *in vivo* experiments demonstrate that PBO exerts potent neuroprotective effects by modulating key inflammatory mediators and pathways. Its actions are primarily mediated through the suppression of NF-κB signaling, with minimal involvement of MAPK pathways. These findings validate the traditional use of PBO as a brain tonic and highlight its potential as a protective product for neurodegenerative diseases.

## Discussion

Our series of studies aimed to provide scientific evidence for polyherbal remedies used as rejuvenating agents, known in Thai as ‘Ya-Ayuwatthana,’ employed by folk healers as a tonic for the brain and entire body [15,16,40,41]. The traditional use of PBO, documented through ethnopharmacological surveys, reflects its cultural significance as a tonic for brain health and overall bodily well-being. This study provides compelling evidence for the first time supporting the neuroprotective effects of PBO against lipopolysaccharide (LPS)-induced neuroinflammation. The findings indicate that PBO significantly mitigates neuroinflammatory responses by modulating the nuclear factor kappa B (NF-κB) signaling pathway, offering a mechanistic basis for its traditional use as a brain tonic and memory enhancer.

The PBO decoction, comprising 11 medicinal plants, was chosen for this study due to its notable antioxidative stress properties. Plant-derived antioxidants are known to neutralize reactive oxygen species, thereby reducing oxidative damage that drives inflammatory responses in cells such as microglia [18,22,26,28,31,38]. Among its herbal components, *P. emblica, T. arjuna,* and *T. bellirica* are primary contributors to the antioxidant capacity of PBO, and previous studies confirm that these plants exert antioxidative, anti-neuroinflammatory, and neuroprotective effects in both in vitro and in vivo models [42–44]. In line with these reports, our findings identified key chemical markers in PBO with neuroprotective potential, including ellagic acid [18,21,22], rutin [23,26,28,31], chlorogenic acid [29,32], ferulic acid [19,25,27], and caffeic acid [20,24,30]. Reported concentrations of ellagic acid that conferred neuroprotection ranged from 0.01 to 100 μM (≈3 μg/mL to 30 mg/mL) across SH-SY5Y, PC12, and primary murine cortical microglia [18,21,22,38], while rutin demonstrated protective effects in PC12 cells at 25 μg/mL to 3 mg/mL [26,28,31]. The levels of these compounds detected in PBO fall within biologically active ranges, suggesting that the formulation harnesses their pharmacological effects. The observation that the FRAP values of the individual constituents were some two to three times more than those of the PBO extract can be attributed to differences in concentration, extraction efficiency, and phytochemical interactions within the combined formulation. In the PBO extract, potential dilution effects and intercomponent interactions (such as complex formation or competitive antioxidant mechanisms) may reduce the overall measurable reducing power. Also, any possible chemical reactions between the phytochemicals in the mixture could cause antagonistic effects thus, reducing the total measured reducing power, of the combined formulation, of phytochemicals in the mixture, compared to that of the phytochemicals in the mixture. Importantly, the co-presence of ellagic acid, rutin, and other phytochemicals in PBO may act synergistically, enhancing neuroprotection beyond what has been reported for single compounds alone. These chemical markers also provide a valuable quality control measure, reducing batch-to-batch variability while preserving consistent biological activity.

Dysregulation of the inflammatory response can lead to significant damage within the brain, which is associated with the pathogenesis of several neurodegenerative diseases and adversely affects the normal functions of the central nervous system [45]. The prevention of excessive NO production mediated by PBO may be attributed to the inhibition of iNOS, while the diminished overexpression of COX-2 indicates that this herbal formula could suppress the overproduction of PGE_2_. The neuroinflammatory prevention effect of PBO on the mRNA and protein expression of iNOS and COX-2 in brain tissue, induced by LPS, has been robustly validated in mice that consumed PBO consecutively for seven days at doses of at least 100 mg/kg. The underlying anti-inflammatory mechanisms of PBO, characterized by the inhibition of iNOS and COX-2, leading to reduced production of NO and PGE_2_, correspond with its identified chemical markers. Except for caffeic acid, the anti-inflammatory mechanisms of all chemical markers were identified as the inhibition of NF-κB activation, which agrees with our results and has been documented in microglia and various organs [22,32,46,47]. In addition to the chemical constituents reported from PBO, six of the eleven herbal components used in the preparation of PBO have been reported to attenuate lipopolysaccharide-induced NO and PGE_2_ production. These include *P. emblica* [48], *T. bellirica* [49], *Allium sativum* [50], *Cyperus rotundus* [51], *Tinospora crispa* [52], and *Zingiber officinale* [53].

Suppressing the mRNA expressions of inflammatory and neurotoxic cytokines, such as TNF-α, IL-1β, and IL-6, has been proposed as a mechanism for combating chronic inflammation observed when pretreating with PBO, which could potentially reduce neurodegeneration in both microglial cells and the brain. These neuroprotective effects were also observed through the anti-neuroinflammatory activities of ellagic acid, rutin, ferulic acid, chlorogenic acid, and caffeic acid [20,29,30,32,38,47]. Collectively, these compounds could be proposed as chemical markers of PBO that mediate neuroprotective effects by reducing neuroinflammation through the inhibition of NF-κB signaling pathway activation.

Beyond molecular endpoints, the translational potential of PBO lies in its ability to influence behavioral outcomes relevant to neurodegenerative diseases. Preclinical studies of its key compounds report reversal of cognitive deficits, improved motor coordination, and reduced behavioral despair in models of Alzheimer’s and Parkinson’s disease [23–25,27,29]. These findings support the rationale that PBO, by jointly targeting oxidative stress and NF-κB-driven neuroinflammation, may delay disease progression. Therefore, the ability of PBO to attenuate the mRNA and protein expression of iNOS and COX-2, subsequently affecting the release of their respective end-products, NO and PGE_2_, along with its inhibitory effect toward the overexpression of TNF-α, IL-1β, and IL-6, could be beneficial in preventing and delaying the progression of neurodegenerative diseases. Nevertheless, there are still few gaps: Genetic diversity plays a very important role in explaining the variation in response of people to treatments, the rate at which drugs are metabolized and the therapeutic outcomes obtained. Genetic variations in the regulation of drug-metabolizing enzymes, transporters, and target receptors have the potential to change the bioavailability and efficacy of plant-based formulations including PBO. The pharmacodynamics and pharmacokinetics of bioactive compounds can also be altered by ethnicity-related factors such as diet, gut microbiome, and environmental exposures. Though the results provide valuable information on the effectiveness of PBO, they might not be generalized to other populations. Further research needs to adopt a sample population with a wide range of genetic and ethnic differences, so as to demystify and substantiate the variation that exists and enhances the translational application of PBO as a complementary therapeutic agent among different population groups. Furthermore, PBO itself has not been tested behaviorally in animal models, its constituents have not been studied pharmacokinetically and bioavailability, and its safety under chronic neuroinflammatory conditions has not been assessed. More importantly, its efficacy and tolerability in humans need to be proven by clinical validation. Overcoming these shortcomings will define whether PBO will be able to shift to an evidence-based therapeutic use beyond ethnomedicine.

## Conclusion

This study provides the first experimental validation of the neuroprotective effects of Phy-Blica-O (PBO), a traditional Thai polyherbal formulation long used as a brain tonic. PBO demonstrated significant inhibition of LPS-induced neuroinflammation in both cellular and animal models by suppressing the NF-κB signaling pathway and reducing the expression of iNOS, COX-2, and pro-inflammatory cytokines (TNF-α, IL-1β, IL-6). These results substantiate its ethnomedicinal reputation and indicate its potential as a cost-effective multi-target therapeutic candidate for preventing neurodegenerative diseases associated with inflammation. However, this study did not evaluate behavioral outcomes, pharmacokinetics, or long-term safety, and the bioavailability and interactions among PBO’s multiple constituents remain unclear. Future research should focus on pharmacokinetic and toxicological assessments, behavioral testing in cognitive and motor models, and investigations using chronic neurodegeneration models to establish its disease-modifying potential. Standardization of its bioactive components and well-designed clinical trials are also essential to confirm efficacy, safety, and optimal dosage in humans.

## Supporting information

S1 Fig Graphical Abstract. (DOCX)

S1 Dataset for Figures. (XLSX)

S2 Dataset for Tables. (XLSX)

S1 Table Ingredients and proportions of the Phy-Blica-O decoction (previously published as THP-R016), as described by Mr. Yop Lomsa, a folk healer from Phattalung Province, are utilized as a body tonic rejuvenator. (DOCX)

## Abbreviations

ABTS: 2,2’-Azino-bis(3-ethylbenzothiazoline-6-sulfonic acid)
AD: Alzheimer’s disease
COX-2: Cyclooxygenase-2
DPPH: 1,1-diphenyl-2-picrylhydrazyl
FRAP: Ferric reducing antioxidant power
HPLC: High-performance liquid chromatography
Inos: Nitric oxide synthase
IL-1β: Interleukin 1β
LMICs: Low- and middle-income countries
LPS: Lipopolysaccharide
MAPK: Mitogen-activated protein kinase
MCI: Mild cognitive impairment
NBT: Nitroblue tetrazolium
NF-κB: Nuclear factor kappa B
NO: Nitric oxide
ORAC: Oxygen radical absorbance capacity
PGE_2_: Prostaglandin E2
TLR4: Toll-like receptor 4
TNF-α: Tumor necrosis factor α
TPTZ: 2,4,6-Tris(2-pyridyl)-s-triazine
