# Supplementary figures and images for: Neuroprotective mechanisms of Thai traditional brain tonic Phy-Blica-O against LPS-induced neuroinflammation: Inhibition of NF-κB in microglia and mice
Source: PLoS One. 2026 Jun 26;21(6):e0352429. doi: 10.1371/journal.pone.0352429 (PMC13308771; doi:10.1371/journal.pone.0352429)

**Graphical Abstract**


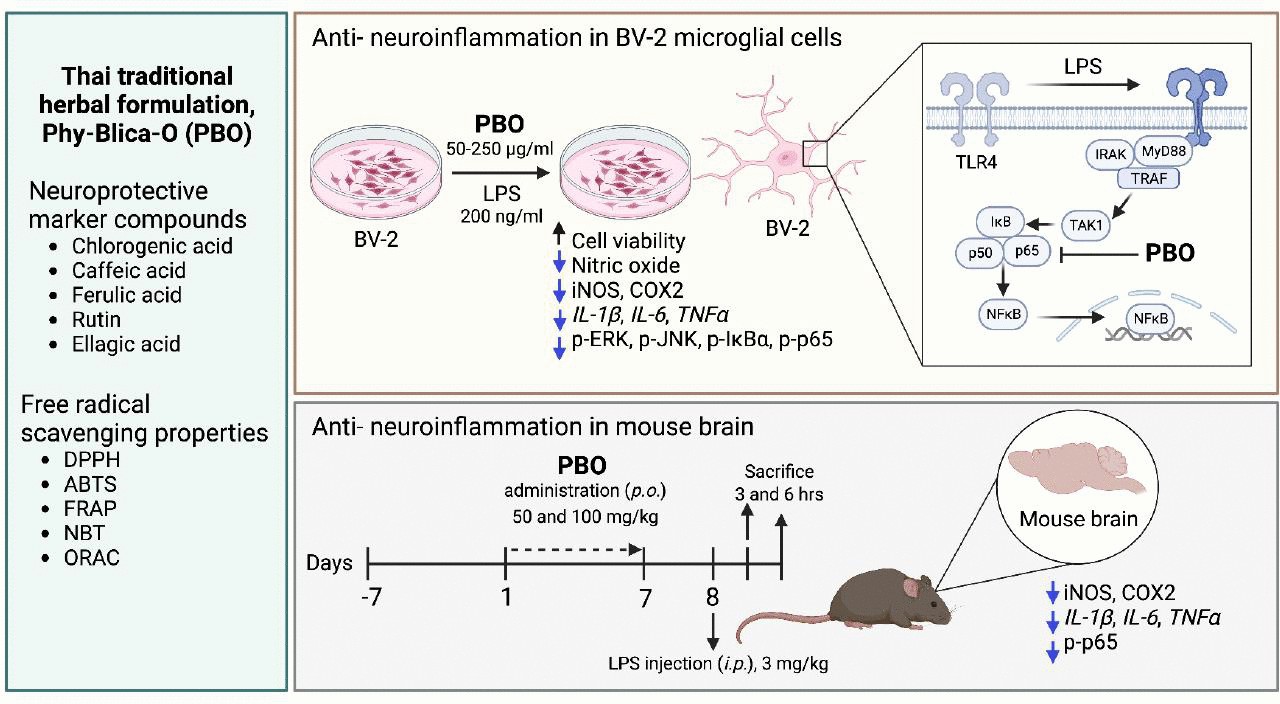

Supplement: S1 Fig — (DOCX) [file pone.0352429.s001.docx]
