# Supplementary material for: Neuroprotective mechanisms of Thai traditional brain tonic Phy-Blica-O against LPS-induced neuroinflammation: Inhibition of NF-κB in microglia and mice
Source: PLoS One. 2026 Jun 26;21(6):e0352429. doi: 10.1371/journal.pone.0352429 (PMC13308771; doi:10.1371/journal.pone.0352429)
Supplement: S1 Table — (DOCX) [file pone.0352429.s004.docx]

**Supplementary Table S1** Ingredients and proportions of the Phy-Blica-O decoction (previously published as THP-R016), as described by a folk healer from Phattalung Province, are utilized as a body tonic rejuvenator.

Herbal components Family Parts used Materia medica

Voucher No.

*Allium sativum* L. Amaryllidaceae Bulb MTM08-04

*Alpinia galanga* (L.) Willd. Zingiberaceae Rhizomes MTM08-05

*Cyperus rotundus* L. Cyperaceae Rhizomes MTM08-33

*Maerua siamensis* (Kurz) Pax Capparaceae Root MTM08-57

*Phyllanthus emblica* L. Phyllanthaceae Fruit MTM08-72

*Piper retrofractum* Vahl Piperaceae Fruit MTM08-79

*Terminalia arjuna* (Roxb. Ex DC.) Combretaceae Fruit MTM08-90

Wight & Arn.

*Terminalia bellerica* (Gaertn.) Roxb. Combretaceae Fruit MTM08-91

*Terminalia citrina* (Gaertn.) Combretaceae Fruit MTM08-93

Roxb. ex Fleming

*Tinospora crispa* (L.) Menispermaceae Stem MTM08-95

Hook. f. & Thomson

*Zingiber officinale* Roscoe Zingiberaceae RhizomesMTM08-98

According to previously published data (Chanthasri et al. 2021; Chanthasri et al. 2018), the taxonomic identities of the medicinal plant samples utilized in this study have been authenticated and verified, and their voucher specimens have been deposited in the Herbarium of Materia Medica at the Faculty of Traditional Thai Medicine, Prince of Songkla University, Thailand.
